# Effects of Glucagon-Like Peptide-1 Receptor Agonists on Body Weight: A Meta-Analysis

**DOI:** 10.1155/2012/672658

**Published:** 2012-05-20

**Authors:** Matteo Monami, Ilaria Dicembrini, Niccolò Marchionni, Carlo M. Rotella, Edoardo Mannucci

**Affiliations:** ^1^Geriatric Cardiology, Careggi Teaching Hospital and University of Florence, 50141 Florence, Italy; ^2^Obesity Agency, Careggi Teaching Hospital and University of Florence, 50141 Florence, Italy; ^3^Diabetes Agency, Careggi Teaching Hospital and University of Florence, 50141 Florence, Italy

## Abstract

Glucagon-Like Peptide-1 receptor agonists (GLP-1RAs), approved as glucose-lowering drugs for the treatment of type 2 diabetes, have also been shown to reduce body weight. An extensive Medline, Cochrane database, and Embase search for “exenatide,” “liraglutide,” “albiglutide,” “semaglutide,” and “lixisenatide” was performed, collecting all randomized clinical trials on humans up to December 15, 2011, with a duration of at least 24 weeks, comparing GLP-1 receptor agonists with either placebo or active drugs. Twenty two (7,859 patients) and 7 (2,416 patients) trials with available results on body weight at 6 and 12 months, respectively, were included. When compared with placebo, GLP-1RAs determine a reduction of BMI at 6 months of −1.0 [−1.3; −0.6] kg/m^2^. Considering the average BMI at baseline (32.4 kg/m^2^) these data means a weight reduction of about 3% at 6 months. This result could seem modest from a clinical standpoint; however, it could be affected by many factors contributing to an underestimation of the effect of GLP-1RA on body weight, such as non adequate doses, inclusion criteria, efficacy of GLP-1RA on reducing glycosuria, and association to non-pharmacological interventions not specifically aimed to weight reduction.

## 1. Introduction

Most drugs developed for the therapy of obesity have failed to show a sufficient efficacy and safety for long-term treatment. In particular, agents which stimulate energy expenditure (e.g., thyroid hormones, sympathoadrenergic drugs, or sibutramine) do not have an adequate cardiovascular safety, whereas centrally acting anorexants either are ineffective in the long term (e.g., serotonin reuptake inhibitors) or show neuropsychiatric adverse effects (e.g., amphetamine derivatives or cannabinoid receptor antagonists) [1]. As a result, orlistat, which inhibits lipid absorption, is the only available drug for obesity in many countries. Even for drugs which do not show relevant problems of long-term safety, such as orlistat, the unsatisfactory tolerability profile limits clinical use.

Glucagon-like peptide-1 (GLP-1) is a gastrointestinal hormone, produced mainly in the postprandial phase, which stimulates insulin secretion and inhibits glucagon release in a dose-dependent fashion [2]. Due to this properties, the hormone reduces hyperglycemia without inducing hypoglycemia in patients with type 2 diabetes [3]. The rapid inactivation of GLP-1 in vivo and the consequent short half-life (a few minutes after subcutaneous administration) prevents its therapeutic use. Long-acting GLP-1 receptor agonists, which can be administered via subcutaneous injection once or twice a day or once a week, have been developed as glucose-lowering drugs for the treatment of type 2 diabetes [4], but they have also been shown to reduce body weight [5, 6]. The effects of GLP-1 and its agonists on body weight appears to be due to a reduction in food intake, mainly determined by a direct central (hypothalamic) effect of the hormone [7]. The stimulation of GLP-1 receptor also retards gastric emptying; this latter effect is again due, at least partly, to a central action, mediated via the autonomous nervous system [8]. One of the side effect of GLP-1 receptor agonists, nausea (sometimes associated with vomiting), could contribute to the weight reducing effect; however, weight loss has also been observed when analyzing separately patients who do not report nausea [8].

In fact, some drugs of this class (i.e., liraglutide and long-acting exenatide) are currently under development for the treatment of obesity [9–12]. A phase II, 20-week trial enrolling patients without diabetes showed that liraglutide has a higher efficacy than orlistat in promoting weight loss [13]. Another longer-term (52 weeks) trial with same molecule, the results of which have not been published in full but partly disclosed [14], confirms that liraglutide is an interesting option for the treatment of obesity. Another molecule of the same class, exenatide, has been reported to induce a significant weight loss in a 24-week placebo-controlled trial [15]. Most of what is known on the effect of GLP-1 receptor agonists on body weight comes from clinical trials performed on patients with type 2 diabetes, with glucose control as the principal endpoint. Currently ongoing trials enrolling subjects with obesity and without diabetes will provide, in due time, further information. In the meanwhile, a systematic evaluation of data collected in studies on type 2 diabetes can provide a more defined picture of what we can realistically expect from GLP-1 receptor agonists as weight-reducing agents.

A recent meta-analysis has shown a weight loss of approximately 3% at endpoint in available published trials, with a duration ranging from 20 to 52 weeks [6]. This analysis does not provide information on the time-course of weight loss with GLP-1 receptor agonists. Furthermore, no distinction is made between placebo- and active comparator-controlled trials, with some of the comparators (i.e., insulin, thiazolidinediones, and sulfonylureas) possibly inducing weight gain. Aim of the present meta-analysis is to assess the effects of GLP-1 receptor agonists on body weight at 6 and 12 months of treatment, separating placebo-controlled trials from comparisons with active drugs. Furthermore, a meta-regression analysis will be performed to explore predictors of weight change during treatment.

## 2. Methods

The meta-analysis was reported following the PRISMA checklist [16].

### 2.1. Data Sources, Searches, and Extraction

An extensive Medline, Cochrane database, and Embase search for all articles in English using the keywords “exenatide”, “liraglutide”, “albiglutide”, “semaglutide”, and “lixisenatide” was performed collecting all randomized clinical trials on humans up to December 15, 2011. Completed but still unpublished trials were identified through a search of http://www.clinicaltrials.gov/ website. FDA (http://www.fda.gov/) and European Medicines Agency (EMA, http://www.ema.europa.eu/) reviews of approved drugs, as well as published information provided to FDA in response to queries during the approval process, were also searched for retrieval of unpublished trials. Results of those trials were retrieved, if available, on http://www.novonordisk-trials.com/ or http://www.clinicaltrials.org/. For unpublished and published trials which were not exhaustively disclosed, an attempt was made (through e-mail) to contact principal investigators in order to retrieve missing data. For all published trials, results reported in papers were used as the primary source of information, when available.

The identification of relevant abstracts, the selection of studies based on the criteria described previously, and the subsequent data extraction were performed independently by two of the authors (E. Mannucci, M. Monami), and conflicts these resolved by the third investigator (N. Maschionni).

### 2.2. Study Selection

A meta-analysis was performed including all randomized clinical trials, with a duration of at least 24 weeks, comparing full therapeutic doses Glucagon-like Peptide-1 (GLP-1) receptor agonists (i.e., at least 1.8 mg/day liraglutide, 20 *μ*g/day for exenatide b.i.d., 2 mg/day for exenatide once weekly) and with placebo or other active drugs. Trials with a shorter duration were excluded, due to the fact that they could not yield relevant information on body weight reduction. No review protocol was published elsewhere. Trials without any information on body mass index (BMI) at 6 or 12 months were also excluded.

### 2.3. Quality Assessment

The quality of trials was assessed using some of the parameters proposed by Jadad et al. [17]. The score was not used as a criterion for the selection of trials, whereas some items were used only for descriptive purposes.

### 2.4. Data Synthesis and Analysis

The principal outcome was the effect of full therapeutic doses of GLP-1 receptor agonists, compared with other hypoglycemic agents or placebo, on BMI at 6 months and 12 months (when available). Between-group differences in endpoint BMI were assessed as a measure of treatment effect, without considering differences from baseline. Secondary outcomes included glycated hemoglobin (HbA1c) at 6 and 12 months. Separate analyses were performed for trials with different GLP-1 receptor agonists and with different comparators, whenever possible. Furthermore, separate analyses were performed for trials with different principal endpoints. Metaregression analysis was performed on placebo-controlled trials, in order to identify possible predictors of weight loss.

Heterogeneity was calculated using the *I^2^* statistics. Weighted mean differences were calculated for BMI and HbA1c at 6 and 12 months, and a random effects model was used for the meta-analysis. Publication/disclosure bias was estimated separately for placebo-controlled trials and studies versus active comparators, using Kendall's tau without continuity correction, and one-sided *P*, were calculated, together with the fail-safe *N*, and Funnel plot analysis. All those analyses were performed using Comprehensive Meta-analysis Version 2, Biostat, (Englewood, NJ, USA). 

## 3. Results

**Figure 1:**
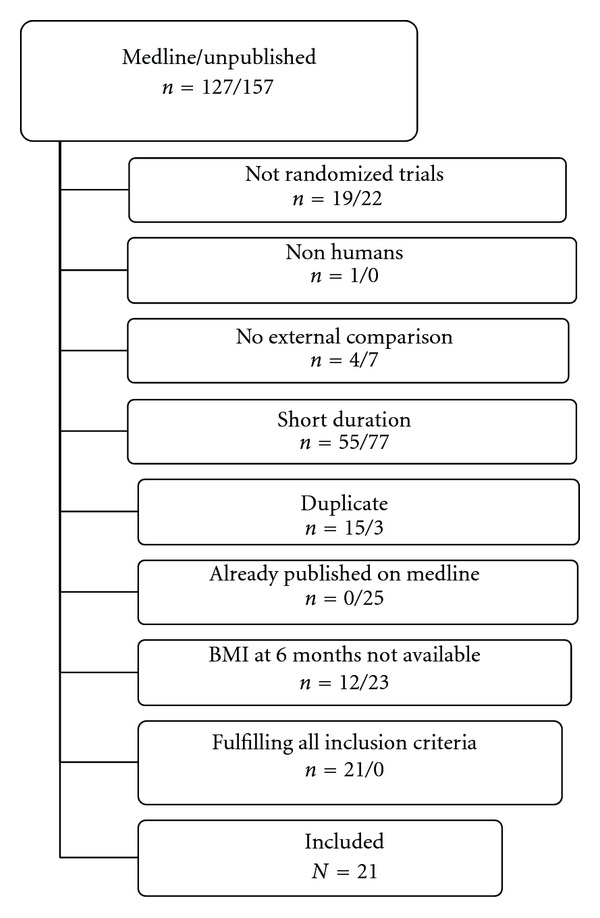
Trial flow summary.

**Figure 2:**
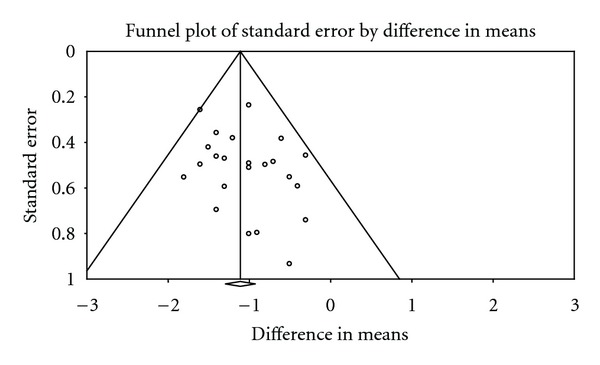
Funnel plot for bias/disclosure publication.

The trial flow summary is reported in Figure 1. Trials with available results on body mass index at 6 months were 21 (19 of which in patients with diabetes), whereas those with data at one year were 7 (6 of which on diabetes); the characteristics of those studies are summarized in Table 1. Funnel plot analysis on 6-month trials on diabetes (Figure 2) did not reveal any major publication/disclosure bias for BMI, as confirmed by Kendall's tau (*t* = 0.14, *P* = 0.36) and fail-safe *N* (number of missing studies that would bring *P* > 0.05: 733). *I^2^* for BMI at 6 months was 83.6 (*P* < 0.001).

### 3.1. Results at 6 Months

Only two trials [15, 18] in subjects with obesity not associated with diabetes reported outcomes on body weight of exenatide at 6 months (with a significant (*P* = 0.002) BMI reduction of 1.6 (0.6–2.5) kg/m^2^ in comparison with placebo). One further trial, which enrolled patients with type 2 diabetes, had been designed for the assessment of weight reduction with exenatide as the principal outcome [19], with similar results.

**Figure 3:**
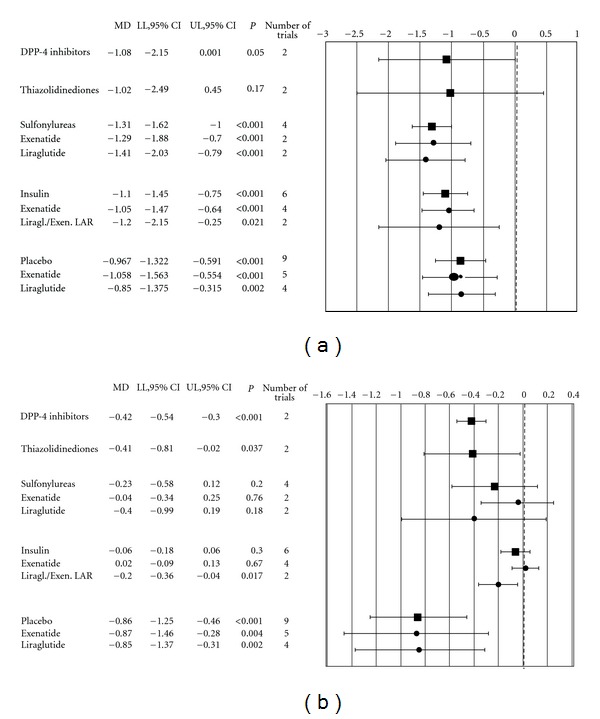
Weighted mean differences in 6-month BMI (a) and HbA1c (b) between GLP-1 receptor agonists and different active comparators or placebo, in trials performed in type 2 diabetic patients. MD: weighted mean differences; LL: lower limits; UL: upper limits.

In the 19 trials performed in patients with diabetes, GLP-1 receptor agonists were associated with a significantly lower BMI at 6 months in comparison with placebo and with any active glucose-lowering agent, with the exception of the only 2 available head-to-head comparisons with thiazolidinediones. No differences in the weight-reducing effects were observed between exenatide and liraglutide (Figure 3(a)). A subgroup analysis of placebo-controlled trials was performed on the basis of the minimum BMI chosen as inclusion criterion; in trials excluding (*N* = 4) or including (*N* = 5) nonoverweight (BMI < 25 kg/m^2^), the difference in 6-month BMI between active treatment and control groups was −1.0 [−1.6; −0.4] and −0.8 [−1.3; −0.3] kg/m^2^, respectively (both *P* < 0.001).

For 18 out of 19 of those trials, the principal endpoint was HbA1c, which was significantly reduced by GLP-1 receptor agonists in comparison with placebo, DPP4 inhibitors, and thiazolidinediones, whereas differences with respect to sulfonylureas and insulin were not statistically significant (Figure 3(b)).

Metaregression analysis was performed on all placebo-controlled trials, including those on nondiabetic individuals, irrespective of the principal endpoint of the study. In the 11 available trials, mean baseline BMI, age, and duration of diabetes (in the 9 trials on patients with diabetes) were not significantly correlated with treatment effect on BMI.

### 3.2. Results at 12 Months

Results on BMI at 12 months were available in 7 trials, 6 of which were performed in patients with diabetes. The only one trial [14] enrolling subjects without diabetes, which had weight loss as its principal endpoint, liraglutide, induced a significant reduction of weight in comparison with placebo (−1.2 [−2.3; −0.1] kg/m^2^ in 1-year BMI; *P* = 0.04). The results of the other 6 trials, all with active comparators, are summarized in Table 2. In these studies, a further reduction of body weight was observed after the first six months of treatment. Similar results were obtained when the only trial which did not report 6-month BMI [14] was excluded from the analysis (data not shown).

## 4. Discussion

The few available trials designed with weight loss as the principal endpoint and enrolling nondiabetic patients with obesity have shown that GLP-1 receptor agonists have a potential use as drugs for the treatment of overweight [14, 15]. Similar results were obtained in a trial on overweight patients with polycystic ovary syndrome, in which restoration of menstrual cycles was the principal endpoint [20]. The much wider evidence collected in subjects with type 2 diabetes confirms this effect, as previously reported [6, 18]. This action is consistent across trials, and it cannot be attributed to selective reporting as shown by Funnel plot analysis and Kendall's tau calculation. GLP-1 receptor agonists have a beneficial effect on body weight not only in comparisons with drugs that induce weight gain (such as insulin or sulfonylureas) but also with respect to placebo. The only exception is represented by direct comparisons with thiazolidinediones: in this case, despite a mean difference in 6-month BMI similar to that observed for other active comparators, the statistical significance is not reached, due to the small number of available trials.

In order to evaluate the weight reducing effect of GLP-1 receptor agonists, the most interesting results are those obtained in placebo-controlled trials, which allow to discriminate the beneficial action of these drugs from the adverse effects on body weight of other glucose-lowering agents. In these studies, the mean weight loss at 6 months is 1.0 kg/m^2^; considering that the average BMI at baseline is about 33.9 kg/m^2^, this means that the actual ponderal reduction is in the 3% range. The estimated weight loss seems to be larger than that reported in a previous meta-analysis [6]; this result could be due to the exclusion of patients treated with submaximal doses of GLP-1 receptor agonists. This result could seem modest from a clinical standpoint; however, several factors should be considered. In all trials on patients with diabetes except one the principal endpoint was the improvement in HbA1c, and not weight loss. This means that patients were selected on the basis of unsatisfactory glucose control, and not for their overweight; the minimum BMI for inclusion was not specified in some studies, and ranged from 25 to 45 kg/m^2^ in the others, meaning that, in all trials, part of the patients enrolled were not actually obese. Notably, those trials that excluded normal-weight subjects showed a greater effect of GLP-1 receptor agonists on weight loss. Furthermore, patients with diabetes could have greater difficulties in losing weight than similarly overweight subjects with normal glucose tolerance. In those who had elevated HbA1c at baseline, the reduction of glycosuria determined by drug treatment could have been an obstacle to weight loss. Finally, the nonpharmacological interventions associated to drugs in trials for glycemic control in type 2 diabetes are not specifically aimed at weight reduction. All these factors could have contributed to an underestimation of the effect of GLP-1 receptor agonists on body weight. It should also be recognized that weight loss in clinical trials could be quite different from that obtained in real-life conditions. The selection of patients with greater compliance and the more accurate follow-up produces a greater weight loss from baseline in randomized clinical trials. On the other hand, for the same reasons, as long as the between-group differences are assessed, as in the present study, the lifestyle/dietary intervention associated with drug treatment in randomized trials can partly mask the actual effect of the drug.

It should also be considered that treatment with GLP-1 receptor agonists could have some further beneficial effects on other metabolic alterations of obese patients (e.g., insulin resistance, risk of diabetes, blod pressure, etc.), beyond weight loss. The assessment of those effects was not among the aims of the present meta-analysis.

The effect of GLP-1 and its receptor agonists on food intake and body weight is dose dependent [13]. For this reason, it is possible that doses needed for the treatment of obesity are higher than those indicated for type 2 diabetes. For example, liraglutide 3.0 mg/day induces a greater weight loss than 1.8 mg/day, whereas no additional effect on blood glucose is expected over 1.8 mg/day [13]. Obviously, at least some of the adverse effects of these drugs (e.g., nausea and vomiting) are also dose-dependent and they could be amplified by the increase in daily doses. In the case that recommended doses for obesity exceed in a relevant manner those for diabetes, the safety profile of GLP-1 receptor agonists, which is satisfactory when they are used in the treatment of type 2 diabetes, should be verified on a sufficiently wide amount of data.

Some interesting information can be obtained from the analysis of data collected in trials on type 2 diabetes with a 1-year follow-up; in fact, the effect of GLP-1 receptor agonists at 1 year seems to be larger than that observed, in the same trials, after 6 months of treatment. The number of studies is limited, and none of them includes a comparison with placebo; in fact, a longer-term treatment without any active drug would be unethical in patients with unsatisfactory control of diabetes. Active comparisons can be misleading, as the comparators often induce weight gain (e.g., insulin, sulfonylureas, thiazolidinediones); this means that the increased difference between GLP-1 receptor agonists and control groups at 1 year could be partly due to weight gain induced by comparators. Despite these limitations, the possibility that the maximum effect of GLP-1 receptor agonists on body weight is reached after 6 months should be considered and taken into account in the design of future clinical trials.

## Figures and Tables

**Table 1:** 

Study (Reference)	Number of patients (ID/C)	Comparator	Trial duration (wks)	Age (ys)	Duration of DM (ys)	BMI Baseline (Kg/m^2^)	BMI at 6-month (Kg/m^2^)	HbA1c baseline (%)	HbA1c at 6-month (%, ID/C)
*Liraglutide *									
Marre et al. [21]	234/114	Placebo	26	56	7.0	30.0	29.9/30.3	8.5	7.5/8.7
Nauck et al. [22]	242/121	Placebo	26	57	8.0	31.3	30.1/31.1	8.3	7.3/8.5
Zinman et al. [23]	355/175	Placebo	26	55	9.0	33.5	33.0/33.7	8.5	7.0/7.9
Russell-Jones et al. [24]	230/114	Placebo	26	57	9.0	30.9	29.9/31.2	8.3	7.0/8.1
Nauck et al. [22]^#^	242/242	Glimepiride	26	57	8.0	31.1	30.1/31.6	8.3	7.3/7.4
Garber et al. [25]	498/248	Glimepiride	52	53	5.0	33.0	32.3/33.6	8.3	7.1/7.8
Marre et al. [21]^#^	234/232	Rosiglitazone	26	56	7.0	29.8	29.9/30.2	8.4	7.5/8.1
Pratley et al. [26]	225/219	Sitagliptin	52	55	6.0	32.8	29.9/31.5	8.4	6.9/7.3
Russell-Jones et al. [27]^ #^	230/232	Glargine	26	57	9.0	30.3	29.9/30.9	8.3	7.0/7.2

*Exenatide LAR*									
Diamant et al. [27]	233/233	Glargine	26	58	8.0	32.0	31.1/32.5	8.3	6.8/7.0
Bergenstal et al. [28]	160/165	Pioglitazone	26	52	6.0	32.0	31.2/33.0	8.5	7.2/7.4
Bergenstal et al. [28]^#^	160/166	Sitagliptin	26	52	6.0	32.0	31.2/31.7	8.5	7.2/7.7

*Exenatide*									
Obesity									
Rosenstock et al. [15]	73/79	Placebo	24	46	0.0	39.5	37.8/38.8	5.6	5.6/5.7
Elkind-Hirsch et al. [20]	20/20	Placebo	24	29	0.0	40.4	39.1/40.8	NR	NR/NR
Type 2 diabetes									
Moretto et al. [29]	77/78	Placebo	24	54	2.0	31.5	30.4/31.4	7.8	6.9/7.6
Buse et al. [30]	129/123	Placebo	30	55	6.3	33.3	32.4/33.8	8.6	7.8/8.7
Buse et al. [31]	138/123	Placebo	30	59	12.0	33.5	33.2/33.5	8.4	6.6/7.5
Kendall et al. [19]	241/247	Placebo	30	55	9.0	34.0	32.9/33.7	8.5	7.7/8.6
DeFronzo et al. [32]	113/113	Placebo	30	53	5.8	34.0	33.0/33.9	8.2	7.4/8.3
Derosa et al. [33]	61/62	Glibenclamide	52	56	NR	28.6	27.3/28.9	8.8	8.1/8.0
Derosa et al. [34]	57/54	Glimepiride	52	55	NR	28.4	27.5/28.5	8.7	7.9/8.1
Gallwitz et al. [35]	248/246	BiAsp	26	57	5.0	33.1	32.0/33.2	7.9	6.9/6.8
Nauck et al. [36]	253/248	BiAsp	52	59	9.9	30.4	29.9/30.5	8.6	7.5/7.6
Heine et al. [37]	282/267	Glargine	26	59	9.5	31.3	30.6/32.0	8.2	7.2/7.1
Bunck et al. [38]	36/33	Glargine	52	58	5.0	30.5	29.9/30.4	7.5	6.7/6.8

^#^Studies with multiple comparators; NR: not reported; ID: interventional drug; C: comparator; DM: diabetes mellitus; wks: weeks.

**Table 2:** Weighted mean differences in 6- and 12-month BMI between GLP-1 receptor agonists and different active comparators.

	Number of trials	6-month BMI	*P*	12-month BMI	*P*
Overall	6	−1.2 [−1.5; −0.8]	<0.001	−1.9 [−3.0; −0.8]	<0.001
DPP-4 inhibitors	1	−1.6 [−2.6; −0.8]	<0.001	−1.7 [−2.7; −0.7]	0.001
Sulphonylureas	3	−1.4 [−2.4; −0.7]	0.001	−2.3 [−4.2; −0.5]	0.012
Insulin	2	−0.7 [−1.4; 0.0]	0.048	−1.5 [−2.1; −0.8]	<0.001
